# Development and psychometric evaluation of perceived clinical nurses’ professional dignity scale: a sequential-exploratory mixed-method study

**DOI:** 10.1186/s12912-023-01543-y

**Published:** 2023-10-20

**Authors:** Alice Khachian, Abbas Ebadi, Marjan Mardani-Hamooleh, Hosein Bagheri, Ali Abbasi

**Affiliations:** 1 Nursing and Midwifery Care Research Center, Department of Medical Surgical Nursing, School of Nursing and Midwifery, Iran University of Medical Sciences, Tehran, Iran; 2 Behavioral Sciences Research Center, Lifestyle Institute, Baqiyatallah University of Medical Sciences, Tehran, Iran; 3 Nursing Faculty, Baqiyatallah University of Medical Sciences, Tehran, Iran; 4 Nursing and Midwifery Care Research Center, Department of Psychiatric Nursing, School of Nursing and Midwifery, Iran University of Medical Sciences, Tehran, Iran; 5 Department of Nursing, School of Nursing and Midwifery, Shahroud University of Medical Sciences, Shahroud, Iran

**Keywords:** Dignity, Nursing profession, Clinical nurse, Validity, Psychometrics

## Abstract

**Background:**

In recent years, one of the concepts that has received attention in the nursing profession is professional dignity. On the other hand, there was no proper scale to evaluate this concept. This study aimed to develop and evaluate the psychometric properties of perceived clinical nurses’ professional dignity scale (PCNPDS).

**Methods:**

This exploratory sequential mixed method was developed and implemented in Iran. The study was conducted in two phases; (a) item generation by hybrid concept analysis and (b) item reduction by psychometric evaluation including validity and reliability of the developed scale. Also, the interpretability (ceiling and floor effect), stability (intraclass correlation coefficient), and absolute stability (standard error of measurement) were calculated.

**Results:**

68 items in the primary item pool were finally reduced to 22 items after evaluating the validity (face, content, and construct validity) and reliability. Exploratory factor analysis revealed three factors (organizational dignity, dignity-based competency, and dignity-based appreciation) and explained 47.55% of the total extracted variance. Confirmatory factor analysis showed that the model had a good fit. Finally, Cronbach’s alpha coefficient, McDonald’s omega, ICC, and SEM were calculated as 0.90, 0.89, 0.96, and 1.91, respectively.

**Conclusion:**

The 22-item developed scale is valid and reliable for professional dignity measurement among Iranian clinical nurses.

**Supplementary Information:**

The online version contains supplementary material available at 10.1186/s12912-023-01543-y.

## Background

Dignity is important in human rights because it represents an individual’s intrinsic worth [1]. Furthermore, one’s social status can influence their sense of dignity in society [2]. The concept of dignity is divided into two categories: intrinsic and societal. Every individual has intrinsic dignity that is anchored in their inner values and is an essential component of their existence [2, 3]. Social dignity, on the other hand, can be influenced and impacted by a variety of social variables and moral behaviors [2, 4].

The concept of professional dignity in the nursing profession has drawn significant attention and has been subject to investigation in recent years [5–7]. According to Sabatino et al. (2014), in a meta-synthesis, the concept of nurses’ professional dignity is characterized by multiple dimensions. The development of a comprehensive concept is contingent upon various factors, including social and cultural contexts. Some traits are innate to the human condition, whereas others are influenced by an individual’s personal principles, ethical standards, and professional. Furthermore, certain traits may be subject to the impact of the surrounding milieu and organizational culture [8].

It is critical to evaluate the cultural aspects that may influence dignity and professional values in various countries [2]. Combrinck et al. claimed that administrators, health team members, and nurses must highlight and encourage nurses’ professional dignity [6]. According to Abbasi et al. (2023), to promote the professional dignity of nurses, it is recommended to identify the factors threatening their professional dignity and create healthy work environments for them [9].

Stievano et al. suggested that further research in various clinical settings and countries would be beneficial in enhancing our comprehension of the notion of the professional dignity of nurses. Measuring professional dignity in nurses may also have a beneficial impact on patients [10]. However, recent studies did not account for a tool to measure the concept of professional dignity among clinical nurses. While creating a tool with complete psychometric properties can be challenging and time-consuming, the most effective tools are those that have undergone a thorough and precise psychometric evaluation. In recent years, nurses have utilized the principles and foundations of psychometrics to design and test important measurement tools in the field of nursing. So, accurate measurement tools are crucial to ensuring the validity and reliability of research. Adherence to these principles holds significant value for nurse researchers [11]. The instruments available to measure dignity may need to address the concept of professional dignity for nurses fully. These instruments may focus on measuring patients’ perspectives on dignity preservation or evaluating nurses’ perspectives on preserving patients’ dignity [12–14].

There are currently only two instruments for measuring workplace dignity. The first was developed in 2019 by Thomas and Lucas in the United States, while Tiwari and Sharma created the second in India during the same year. The workplace dignity scale (WDS) is a tool used by managers to assess the level of dignity in the work environment for office workers. Its purpose is to enhance the quality of work life for employees [15, 16]. It is important to note that measuring the professional dignity of nurses may require a specialized tool that differs from those used in other professions. Rocco et al.‘s annual scientific report (2020) outlines their plans for developing the Nursing Professional Dignity Scale (NPDS). As of now, there has yet to be any publication regarding the results of this study, including details on item generation, the scale’s dimensions, the number of items, and other related information [17]. Unfortunately, we do not have access to the current scale’s information.

Developing an accurate and comprehensive tool to assess the professional dignity of clinical nurses in Iran is crucial. This tool must be designed and constructed in a way that aligns with Iranian nurses’ unique mentality and working conditions while also adhering to rigorous scientific methodology. Developing effective tools in this area allows for a more organized and thorough exploration of this concept by researchers and nursing managers. Therefore, we utilized a hybrid approach to design and psychometrically measure the perceived professional dignity of clinical nurses.

## Methods

### Study design

**Fig. 1:**
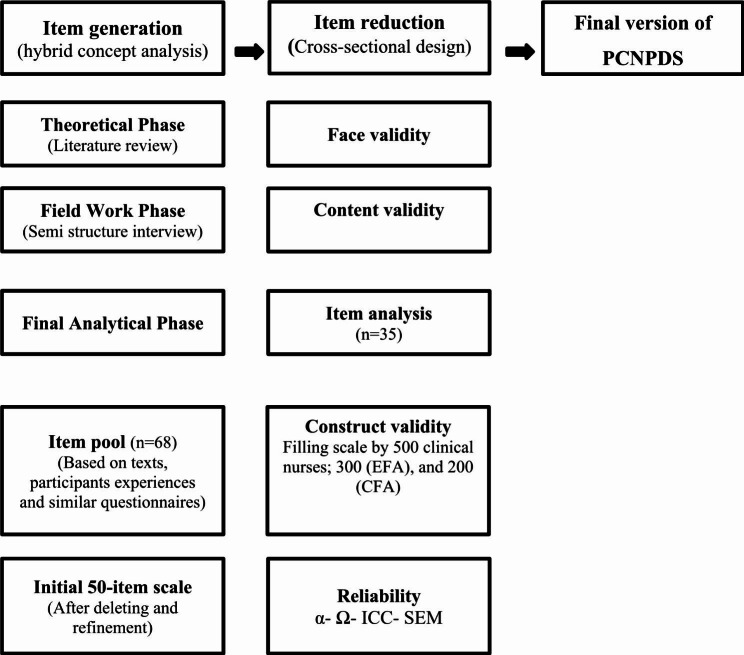
Flow diagram of the development and psychometric Evaluation of the PCNPDS

This sequential exploratory mixed-method study of the instrument-development variant was conducted in Iran among clinical nurses between October 2020 and September 2022. This study consisted of two phases: item generation using a hybrid concept analysis and item reduction using a cross-sectional design to assess the psychometric properties of the generated scale (Fig. 1).

### Item generation

This study utilized a hybrid concept analysis approach, consisting of three stages (theoretical, fieldwork, and final analysis), to explore the perceived professional dignity of clinical nurses and identify its indicators and characteristics [18, 19]. It should be noted that part of the data obtained from the fieldwork stage has been published in Nursing Open [9].

A scoping review was used for the literature search in the theoretical phase. Electronic databases such as PubMed, ISI Web of Science, Scopus, Embase, ProQuest, CINAHL Dissertations & Theses, and Persian databases were searched using the keywords “clinical nurse”, dignity”, and “nursing profession” “professional values” with no time restriction.

Inclusion criteria for selecting of the theoretical phase include:

- The words of professional dignity, nurse, and professional dignity of nurse should be in one of the title, abstract, or keywords.
- The articles should be related to journals and authoritative databases of nursing and health sciences.
- At least one of the definitions, antecedents, attributes, and consequences of professional dignity should be mentioned in the full text of the article.

Studies published in a language other than English or Farsi, brief articles such as the editor’s note, and editorial suggestions were excluded.

Search results were reviewed based on titles and duplicate articles were removed. Then, the abstracts of the selected articles were examined in terms of suitability with the study objectives and inclusion and exclusion criteria. Finally, the text of the articles was examined considering the inclusion and exclusion criteria. In all the mentioned stages, the help of two expert consultants in the field of research was taken to review the selection of articles. After eliminating duplicate and irrelevant studies, 15 texts (consisting of two treatises and 13 articles) were chosen from 1511 studies discovered in the initial search. The full text of eligible articles was reviewed in the next phase.

Individual, face-to-face, in-depth, and semi-structured interviews (32 to 75 min) were conducted from October 2020 to March 2021 to understand the concept comprehensively. Purposive sampling was used to choose 15 clinical nurses.

Inclusion criteria in the qualitative phase include:

- Be concerned about the studied concept.
- Have a desire to participate in research.
- Having full-time employment.
- Have at least a bachelor’s degree.

Nurses with clinical work experience of less than one year were excluded from the study.

The interview queries aligned with the characteristics, antecedents, and outcomes formulated during the theoretical phase (Interview guide in the supplementary file 1). A pilot test was conducted by conducting two interviews, based on which the process of main interviews and data analysis began. However, there were no changes in the interview questions. Interviews were conducted with the participants in their free time and in the nurses’ restroom. Every interview was recorded. After transcribing the recorded data, the interview transcripts were analyzed using directed content analysis and MAXQDA Version 10. The directed content analysis by Hsieh and Shannon [20] was used following the study’s purpose and the framework derived from the theoretical phase. In the concluding analysis stage, the results of the fieldwork stage were compared to the data gathered during the theoretical stage’s literature review. The rigor for qualitative data was provided using the criteria of Guba and Lincoln (1985), cited by Polit in 2018 [21], which included four criteria: credibility, conformability, dependability, and transferability. To ensure the validity of the data, the researchers allocated enough time to collect data; nurses were selected from different shifts and wards with different demographic characteristics; the data were analyzed with great accuracy; and other team members cross-checked the findings. The research team members (including three nursing professors and two Ph.D. students) performed a critical review to verify the data’s reliability. An attempt was made to avoid bias in the data analysis phase. Finally, a detailed descriptive report was prepared regarding the data collection, classification, and analysis.

The questionnaire was designed to measure the characteristics of professional dignity as perceived by clinical nurses. In the following stage, the classes that represent the dimensions of the questionnaire were identified. Following that, the items for each dimension were gathered by thoroughly examining existing literature, input from participants, and a review of comparable questionnaires. Ultimately, a pool of 68 items (N = 68) was created. Many of these items were actually different forms of the same concept. Due to the large number of items and the possibility of some of them overlapping with each other, during several meetings of the research team, overlapping and redundant items were merged or eliminated (An example of the item refinement process in the supplementary file 2). The scale was initially developed with 50 items and underwent the psychometric stage.

### Item reduction

The psychometric features of PCNPDS using a five-point Likert response scale ranging from 5: strongly agree to 1: strongly disagree were assessed in terms of face, content and construct validities, and reliability.

### Face validity

The scale was administered to ten clinical nurses during the qualitative face validity stage. The interviews were conducted to evaluate their opinions on the suitability, level of difficulty, relevance, and clarity of the subject matter. During the quantitative face validity stage, a group of ten clinical nurses was requested to evaluate the significance of each item on a 5-point Likert scale. The scale ranged from 1 to 5; five indicated very important, four indicated important, three indicated almost important, two indicated slightly important, and one indicated not important. The impact score for the items was calculated using the following formula: Impact score = Frequency (%) * Importance.

A score of greater than 1.5 was deemed acceptable for each item [22, 23]. At this stage, we removed four items with an impact score of less than 1.5 and merged two. As a result, the total number of items decreased from 50 to 45.

### Content validity

A group of 12 experts was consulted to assess the content validity of the questionnaire—these experts were nursing assistant professors with questionnaire design and management expertise. The experts were tasked with conducting a qualitative review of the questionnaire and providing feedback on various aspects such as grammar, word usage, item placement, and scaling. The items were adjusted after receiving their comments [23].

The items’ content validity ratio (CVR) and content validity index (CVI) were calculated to assess quantitative content validity. The same 12 experts were asked to validate the content and rate the importance of the elements on a scale of 1–3: 1: not necessary, 2: useful but not necessary, 3: necessary [24].

The following formula was used to calculate CVR:

CVR = (ne - [N / 2]) / (N / 2).

N and ne in this formula represent the total number of experts and the number of experts who rate the item as “necessary,“ respectively. Given the number of experts, the minimum allowable CVR is 0.56 [25]. At this point, 13 items were removed (CVR 0.56), and four items were merged in pairs, reducing the total number of items from 45 to 30.

The CVI indicates the significance of the scale’s items. It is calculable for each scale item (I-CVI) and all items (S-CVI). Therefore, we asked the same 12 experts to evaluate the items based on the options “not related,“ “somewhat relevant,“ “relevant but in need of review,“ and “fully relevant” and to assign a score of 1, 2, 3, or 4 for each option. The CVI of each item was calculated by dividing the number of respondents who assigned that item a 3 or 4 by the total number of respondents. Acceptance of the item was dependent on a CVI above 0.79 being deemed adequate. The score between 0.70 and 0.79 was deemed doubtful and required correction and revision. Also, a score below 0.70 was deemed unacceptable and should be eliminated To eliminate the chance effect, modified Kappa was computed for each item (good = 0.60–0.74, and excellent = Kappa > 0.74; 29) [26]. Each item’s CVI and kappa values were acceptable.

### Item analysis

For item analysis, a convenience sampling method was used to select 35 clinical nurses with an average age of 33.64 ± 7.35 years. This analysis aimed to identify any potential issues with the items and calculate the correlation coefficient between them. We excluded items that had a correlation coefficient of less than 0.3. Deleting an item was also recommended if it increased Cronbach’s alpha. At this stage, the scale’s Cronbach’s alpha coefficient was 0.91, indicating high reliability and internal consistency in measuring the professional dignity of clinical nurses [22]. We excluded three items that had a correlation coefficient of 0.3 or less. We merged two items with a correlation coefficient of more than 0.7 and semantic affinity, reducing the total number of items to 26.

### Construct validity

In the construct validity, the participants were selected by convenience sampling method. By referring to the research environment, the purpose of the research and the specifications of the scale were explained, and if they were willing and agreed to participate in the research, the scale was provided to the participants and they completed the scale in their free time.

The construct validity of PCNPDS was evaluated by Maximum Likelihood Exploratory Factor Analysis (MLEFA) with Promax rotation through 300 samples. Kaiser-Meyer-Olkin (KMO) test and Bartlett test were performed for adequacy sampling. KMO values between 0.7 and 0.8 were considered good and values between 0.8 and 0.9 were considered excellent [27, 28]. The presence of an item in a latent factor was determined based on a factor loading of approximately 0.33, which was estimated using the following formula: CV = 5.152÷ √ (n – 2) in which CV was the number of extractable factors and n was the sample size. Next, item subsets less than 0.3 in size were removed from the EFA [29].

CFA was used to evaluate the most common goodness of fit indices for the proposed model concerning acceptable thresholds using maximum likelihood estimation (200 samples). In confirmatory factor analysis (CFA), the use of maximum likelihood (ML) assumes that the observed indicators follow a continuous and multivariate normal distribution [30]. Many references have reported maximum likelihood as one of the most important methods of estimating CFA [28, 30].

The model fit was examined with Root Mean Square of Error of Approximation [RMSEA < 0.08), Comparative Fit Index (CFI > 0.9), Goodness of Fit Index (GFI > 0.9), Adjusted Goodness of Fit Index (AGFI > 0.9), Normed Fit Index (NFI > 0.9), Parsimonious Normed Fit Index (PNFI > 0.5), Incremental Fit Index (IFI > 0.9) and CMIN / DF (< 3) were accepted] [28, 31].

A survey consisting of 26 questions was completed by 500 nurses employed in the clinical departments of four hospitals from December 2020 to September 2022. The participants were informed about the research’s objective, the number of items, the answering method, the estimated time required to complete the questionnaire, the voluntary nature of participation, the assurance of confidentiality and anonymity of the data, the project’s ethical guidelines, the name of the university and department, and the researcher’s contact information, including their name and email address. Table 1 displays the demographic characteristics of the participants.

**Table 1:** The characteristics of study participants (N = 500)

Variables		Number (%)
**Gender**	Male	160 (32)
	Female	340 (68)
**Educational Status**	Bachelor’s Degree	409 (81.8)
	Master’s Degree	91 (18.2)
**Marital status**	Married	343 (68.6)
	Single	145 (29)
	Divorced	12 (2.4)
**Hospital**	Imam Hossein, Shahroud	219 (43.8)
	Bahar, Shahroud	120 (24)
	Firouzgar, Tehran	123 (24.6)
	Hasheminejad, Tehran	38 (7.6)
**Working shift**	Permanent morning	68 (13.6)
	Permanent night	25 (5)
	Morning and evening	43 (8.6)
	Evening and night	17 (3.4)
	Rotating shift	347 (69.4)
		**Mean (SD)**
**Age (years)**		34.42 (8.62)
**Work Experience (years)**		9.78 (8.87)
**Average working hours per month**		189.35 (24.33)

### Reliability

The internal consistency of PCNPDS was evaluated by calculating Cronbach’s alpha (α) and McDonald’s omega (Ω) for each extracted factor. The internal consistency was deemed satisfactory, as evidenced by the α and Ω coefficients exceeding 0.7 [32].

The ICC was used to evaluate the stability. According to the data, the scale has demonstrated a favorable level of stability with an index higher than 0.75 [31]. A subset of 30 clinical nurses completed the questionnaire twice at two-week intervals. As a rule of thumb, researchers should try to obtain at least 30 heterogeneous samples when conducting a reliability study [33]. Additionally, the reliability was assessed using the standard error of measurement (SEM) through the formula: SEM = SDPooled × √1 − ICC). The minimal detectable change (MDC) was also calculated using the formula: MDC = SEM × √2 × 1.96. Furthermore, the Percent minimal detectable change (MDC%) was determined using the formula: MDC%= (MDC ÷ mean) ×100 [34, 35]. Additionally, comprehensibility was assessed by calculating the ceiling and floor effects.

The COSMIN (COnsensus-based Standards for the selection of health Measurement INstruments) checklist was utilized to assess the psychometric properties in this study.

The statistical analysis was conducted using SPSS version 26.0 and LISREL 8.8.

### Normality, outliers, and missing data

Distribution charts and Mahalanobis distance p < 0.001 were used to assess univariate and multivariate outliers. In addition, we ensured that the univariate normality and multivariate normality distributions were appropriately assessed by examining the skewness (with values within ± 3), kurtosis (with values within ± 7), and Mardia’s coefficient (< 8), respectively. In the present study, the data appeared to be relatively consistent with a normal distribution. A missing listwise procedure was employed for the estimation of CFA. Our team tends to lean towards utilizing listwise deletion rather than imputation due to the observed correlation between missingness and non-responses as well as incomplete questionnaires.

### Ethical approval and consent to participate

The Ethics Committee of Iran University of Medical Sciences, Tehran, Iran, approved this study (code: IR.IUMS.REC.1399.810). The required permissions were also obtained from the selected hospitals. Each participant was completely informed about the study protocol and provided a written and informed consent form before taking part in the study. The participants were allowed to leave the study at any time. All participants were assured that recorded interviews would be kept private and results would be reported anonymously.

## Results

### Item generation

After mixing the results of theoretical and field phases of hybrid concept analysis, it has been determined that the concept of perceived professional dignity of clinical nurses can be characterized by four key attributes: a positive public image, appreciation, and visibility, a sense of support from management, and a sense of professional identity within the organization. Additionally, there are five antecedents, including inherent human dignity, professionalism, effective communication with patients and their families, intra-professional communications, and inter-professional communications. Finally, three consequences are associated with this concept, including impacts on the nurse, the patient, their family, society, and the organization’s effects. The initial pool consisted of 68 items categorized into four dimensions: having a favorable public image, appreciation, and visibility, feeling of managerial support, and feeling of professional identity within the organization. The research team carefully assessed and discussed the pool of items in multiple meetings. Some items were removed due to duplication or overlap. After careful consideration, a questionnaire consisting of 50 items was developed and entered the psychometric phase.

### Item reduction

After performing face and content validity, the number of scale items decreased from 50 to 30. Following the item analysis, four items were removed and merged, and the 26-item scale entered the exploratory factor analysis step.

In MLEFA, the KMO value was 0.893, and Bartlett’s test of sphericity was 3200.976 (P < 0.001). In the model, three factors were extracted based on eigenvalues greater than one. As shown in Table 2, the three factors organizational dignity (10 items), dignity-based competence (8 items), and dignity-based appreciation (4 items) together accounted for 47.55% of the total variance.

**Table 2:** Exploratory Factors analysis of the PCNPDS (N = 300)

Factors	Qn. Item	Factor Loading	item communality (h2)	Eigenvalue (λ)	%Variance
Organizational dignity	18- My preferences are highly respected by managers.	0.890	0.767	7.499	31.43
	19 - Managers facilitate the essential conditions for my professional development.	0.856	0.710		
	22- I feel a sense of dignity that my managers are justice oriented.	0.825	0.616		
	16- Managers support me under any circumstances.	0.775	0.620		
	21 - Managers value my professional abilities.	0.752	0.650		
	15- My efforts are being encouraged by the managers.	0.726	0.606		
	23- I feel a sense of dignity for hospital’s fair payment mechanism.	0.651	0.394		
	20– I feel a sense of dignity that managers include me in organizational decision-making.	0.640	0.506		
	17 - I feel a sense of dignity for my managers’ support.	0.596	0.515		
	24- A nurse’s position is so important in workplace.	0.358	0.240		
Dignity based competency	10- Non-nursing colleagues (doctor, physiotherapist, etc.) treat me respectfully.	0.641	0.441	2.408	8.09
	1-According to people, nurses are an important member of health care team.	0.597	0.346		
	3- According to health professionals, nurses are an important member of health care team.	0.582	0.289		
	4- Health professionals consider scientific position for me.	0.570	0.358		
	2- According to people, nurses are scientific and academic people.	0.503	0.243		
	8 - I am treated with respect by patients and their families.	0.485	0.285		
	9- Nursing colleagues treat me with respect.	0.471	0.268		
	5- Nursing colleagues have provided favorable feedback regarding my profession.	0.403	0.257		
Dignity based appreciation	13 - I feel a sense of dignity due to the nursing colleagues’ appreciation.	0.918	0.834	2.047	8.02
	12- I feel a sense of dignity due to the patient and the family’ appreciation.	0.793	0.642		
	14- I feel a sense of dignity due to the managers’ appreciation.	0.760	0.601		
	6- I feel a sense of dignity when the effort and sacrifice of nurses is recognized through the media.	0.407	0.274		

Also, four items were removed due to factor loadings less than 0.3, thus the total number of scale items reached 22.

### Confirmatory factor analysis

**Fig. 2:**
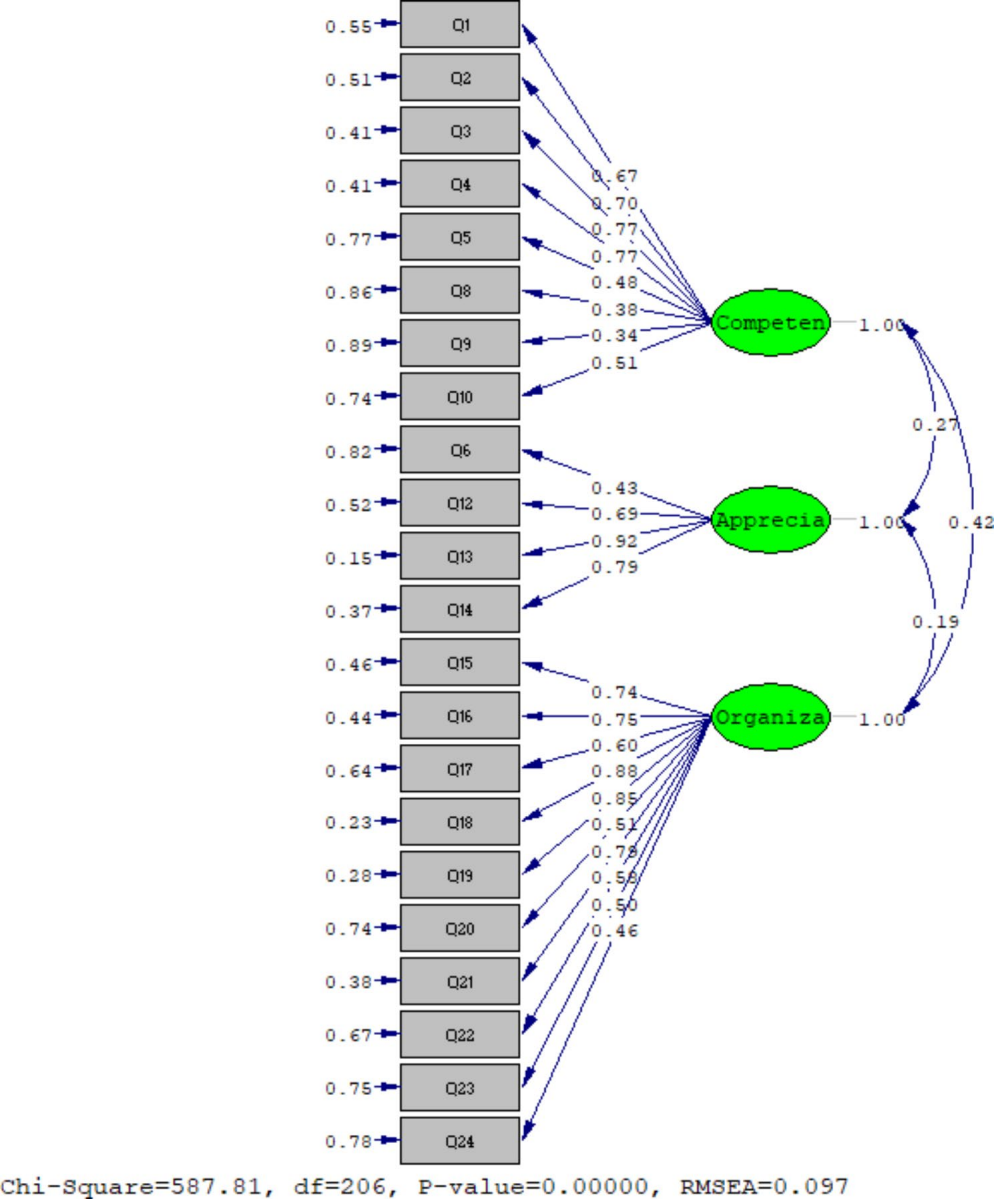
The final model of the PCNPDS (N = 200)

The CFA findings confirmed all the goodness of fit indices of the final model (χ2 = 587.81; DF = 206, P < 0.001, CMIN / DF = 2.84, NFI = 0.86, PNFI = 0.77, RMSEA = 0.097, IFI = 0.91, CFI = 0.91, GFI = 0.79 and AGFI = 0.74) (Fig. 2).

### Reliability

Cronbach’s alpha, McDonald’s omega, and ICC were three excellent factors derived from PCNPDS. Cronbach’s alpha and McDonald’s omega for the 22- item PCNPDS were 0.901 and 0.898, respectively, that indicated excellent internal consistency. The overall ICC was calculated to be 0.96 (CI 95: 0.91–0.98) indicating a strong stability of the scale during the time. The value of SEM for the scale was ± 1.91 which indicated the individuals’ scores on the same scale tend to be distributed 1.91 value around their “true” score. Moreover, absolute reliability based on MDN and MDC% results was 5.27 and 8.09, respectively (Table 3).

**Table 3:** Reliability of the 22- item PCNPDS

Indices	α	Omega	ICC (CI 95%)	SEM	MDC	%MDC
Factor						
Organizational dignity	0.914	0.915	0.95 (0.90–0.97)	1.43	3.95	17.18
Dignity based competency	0.772	0.764	0.93 (0.86–0.97)	1.13	3.12	11.83
Dignity based appreciation	0.812	0.817	0.85 (0.65–0.93)	0.69	1.92	12.19

In addition, the results of the floor and ceiling effects showed that the items are free of these effects and the scale has interpretability.

The final version of the triage nurses’ professional capability questionnaire included 22 items. The score range is between 22 and 110. Answers are scored on a 5-point Likert scale, ranging from 5: strongly agree to 1: strongly disagree (Table 4).

**Table 4:** The final version of the Perceived Clinical Nurses’ Professional Dignity Scale (22 Item)

Item	Strongly agree	Agree	Neither agree nor disagree	Disagree	Strongly disagree
1- According to people, nurses are an important member of health care team.					
2- According to people, nurses are scientific and academic people.					
3- According to health professionals, nurses are an important member of health care team.					
4- Health professionals consider scientific position for me.					
5- Nursing colleagues have provided favorable feedback regarding my profession.					
6- I feel a sense of dignity when the effort and sacrifice of nurses is recognized through the media.					
8- I am treated with respect by patients and their families.					
9- Nursing colleagues treat me with respect.					
10- Non-nursing colleagues (doctor, physiotherapist, etc.) treat me respectfully.					
12- I feel a sense of dignity due to the patient and the family’ appreciation.					
13- I feel a sense of dignity due to the nursing colleagues’ appreciation.					
14- I feel a sense of dignity due to the managers’ appreciation.					
15- My efforts are being encouraged by the managers.					
16- Managers support me under any circumstances.					
17- I feel a sense of dignity for my managers’ support.					
18- My preferences are highly respected by managers.					
19- Managers facilitate the essential conditions for my professional development.					
20- I feel a sense of dignity that managers include me in organizational decision-making.					
21- Managers value my professional abilities.					
22- I feel a sense of dignity that my managers are justice oriented.					
23- I feel a sense of dignity for hospital’s fair payment mechanism.					
24- A nurse’s position is so important in workplace.					

## Discussion

In this study, determining the validation features of the PCNPDS, from the 10 areas related to the COSMIN checklist [36] including validity (content validity, construct validity, and criterion validity), reliability (internal consistency, relative stability, and absolute stability)), responsiveness and interpretability were investigated.

One benefit of the scale was its limited item count, which seems to be helpful in collecting realistic responses given the busy schedules of clinical nurses and their lack of time to complete the questionnaire. The limited item count will lead to more attention and accuracy of the participants in completing the scale. In this regard, Broadbent et al. (2006) stated that the Brief form Questionnaire will be able to repeatedly assessed in a short period of time due to the limited item count and will impose fewer burden on the participants. Also, the participants find this questionnaire easy and scoring will be easier than the long-form questionnaire [37]. Similarly, the workplace dignity scale developed by Thomas and Lucas (2019) with 18 items [15], and the workplace dignity scale by Tiwari and Sharma (2019) with 17 items [16] like the present study, contain the appropriate number of items for effectively assessing workplace dignity.

The following discussion focuses on the three extracted factors of organizational dignity, dignity-based competence, and dignity-based appreciation, based on the factor loading of items in EFA.

The initial factor, organizational dignity, comprised ten items contributing to 31.43% of the total variance. Organizational dignity is the organizational value of nurses, and includes the perception of being supported by managers, justice and equality, and a prestigious position in the organization. This factor is equivalent to the dimensions extracted in the qualitative phase, including a sense of support from management and a sense of professional identity within the organization. In fact, organizational dignity is the perception that nurses have of appropriate feedback and support from managers, appreciation and encouragement, providing conditions for progress and continuous learning, a sense of organizational justice, and the use of competent nurses in important decisions. Also, organizational dignity is the understanding that nurses have of the specific and defined position in the organization and the duties separated from other members of the organization. Also, organizational dignity is equivalent to workplace dignity for nurses. According to Thomas and Lucas, the dimensions of dignity in the workplace include respectful interaction, competence and participation, equality, intrinsic value, public dignity, and abuse or violation of dignity [15]. Respectful interaction is essential in maintaining dignity in the workplace. According to research, respectful interaction is an essential component of dignity, and violations of norms of respect constitute violations of dignity [38, 39]. By respectful interaction and promoting ethics in the workplace, an increase in organizational culture and commitment can be expected [40]. Competence and engagement can be increased through the provision of training and professional development opportunities, the provision of special awards for employees whose performance exceeds expectations, and the establishment of peer recognition programs. Some employees may experience a threat to their dignity as a result of a lack of appreciation for their skills and contributions [15]. According to Tiwari and Sharma, the dimensions of workplace dignity include trust and respect (an individual’s perception of how he is respected and trusted at work), independence (an individual’s perception of freedom of expression and decision-making at work), fair treatment (an individual’s perception of any discrimination, injustice, or unfair treatment), equality (an individual’s perception of equal treatment in the workplace), and self-esteem (an individual’s perception of his or her self-worth) [16], which is equivalent to organizational dignity in this research.

The second extracted factor is dignity-based competence, which consists of eight items and accounts for 8.09% of the total variance. This factor is defined as the nurses’ perception of respect from those around them and the scientific perspective of society and peers regarding their professional competence. In the study conducted by Stievano et al., nurses reported that respectful communication with other healthcare professionals contributed to their sense of dignity [41], resulting in appropriate and mutually respectful relationships with patients. This communication component of dignity, which is emphasized in care ethics [42] and by numerous scientists [43, 44], relates to the social component of dignity. In general, this social recognition provides nurses with great satisfaction and a sense of dignity [41]. Also, dignity-based competence is equivalent to the dimension of having a positive public image, extracted from the qualitative phase. In fact, dignity-based competence is the understanding that nurses have about their profession from the proper perspective of society, patients, colleagues, physicians, and other healthcare professionals. On the other hand, the scientific perspective of the adjacent community plays a significant role in enhancing the sense of professional dignity among nurses. In this regard, in the study by Stievano et al., nurses described that their profession is socially recognized. They also believed that society’s perception of them has always been positive and that they are well-liked [41].

The third factor is dignity-based appreciation and comprises four items, accounting for 8.02% of the total variance. This factor pertains to the level of perception of nurses regarding the importance of appreciation and recognition from society. It is important to acknowledge and value the contributions of nurses in society, as this can enhance their professional esteem and sense of worth, particularly when recognized by patients, families, and colleagues [8]. The dignity-based appreciation factor is equivalent to the appreciation and visibility dimension of the qualitative phase. Being appreciated by family (Sabatino et al., 2014) and being respected by colleagues and others in the clinical environment (Joan Yalden and McCormack, 2010) are examples of appreciation and visibility. Regarding this matter, Stievano et al. found that public health nurses were appreciated by their clients, and some clients expressed a desire to continue receiving care from the same nurses even after relocating to different regions [41]. On the other hand, some nurses have expressed concerns about feeling undervalued and not receiving enough social recognition, which can undermine their professional dignity [2].

In this study, construct validity was consistent with the COSMIN standard. It should be noted that convergent and divergent validity was not performed in this research, which is recommended in future studies. As mentioned, in this research, exploratory factor analysis was performed using the maximum likelihood method. Exploratory factor analysis is used when there is no information available about the construct under study, there is no initial hypothesis about the dimensions of the questionnaire, there is no guess about the structure of the relationships between the items, and the questionnaire was created for the first time [45].

Also in this study, CFA was employed, and fitting of the PCNPDS model was confirmed. Thomas and Lucas utilized both exploratory and confirmatory factor analyses to examine the construct validity of the workplace dignity scale (WDS) in 2019. Through exploratory factor analysis, six factors were extracted. Also, CFA was used and confirmed the WDS model fitness [15]. Also, in the study by Tiwari and Sharma (2019), EFA and CFA were used to determine construct validity. Through EFA, five factors were extracted. The results of CFA indicated the WDS model fitness [16]. In addition, in the study conducted by Scott-Campbell and Williams in 2020, which analyzed the psychometrics of the workplace dignity scale designed by Thomas and Lucas, exploratory factor analysis confirmed the six-factor model, and confirmatory factor analysis demonstrated the model’s good fit [46]. In the study by Kalafatoğlu et al. (2021), who conducted psychometrics of the Turkish version of the workplace dignity scale developed by Thomas and Lucas, only confirmatory factor analysis was used for construct validity, which confirmed the six-factor structure and a good-fitting model of the scale [47].

The scale demonstrated excellent internal consistency in the current study, as evidenced by Cronbach’s alpha and McDonald’s omega results. In the studies on the professional dignity of clinical nurses, there has been limited use of McDonald’s omega factor to evaluate internal consistency. Instead, many studies have relied solely on calculating Cronbach’s alpha coefficient [15, 16, 47, 48]. Also, internal consistency was assessed using Cronbach’s alpha and McDonald’s omega coefficient in a study conducted by Scott-Campbell and Williams [46].

Additionally, this scale has the advantage of demonstrating strong stability, as evidenced by its high ICC value (the accepted standard of the COSMIN checklist). The study utilized a two-week interval between the test and retest stages, which was considered an appropriate timeframe for the test-retest method. Additionally, the participants’ conditions remained stable throughout both stages of completing the scale, further supporting the suitability of this approach [49, 50]. In most of the related studies, the reliability of the scales has not been done based on ICC. Only one study by Kalafatoğlu et al. in 2021 examined the psychometrics of the Turkish version of the dignity of the workplace tool and evaluated the relative reliability using ICC [47].

In this study, the absolute stability was assessed by calculating the standard error of measurement (SEM) for scale. Additionally, the minimum detectable change (MDC) and the percentage of the minimum detectable change (%MDC) were determined and validated according to the COSMIN checklist. Another advantage of this study was the assessment of the PCNPDS’s responsiveness and interpretability. Based on the findings, PCNPDS may have the minimum level of SEM, responsiveness, and interpretability. SEM demonstrates the accuracy of the measurement for each individual and a smaller value is preferred. “Responsiveness” pertains to the capacity of a scale to accurately determine any changes in an individual’s status over a certain duration. Also, interpretability pertains to the scale’s capacity to show the significance of modifications [36]. Previous studies of psychometric characteristics of workplace dignity did not report these particular characteristics.

One of the most important applications of PCNPDS is in nursing policy. Since the professional dignity of nurses is closely related to the quality of care, safety of patients and promotion of organizations, the results of this study can be used in nursing management and policies at different levels. In fact, by using this scale, managers can measure the status of nurses’ professional dignity and formulate policies, also design interventions and evaluate the effectiveness of these interventions in order to maintain the professional dignity of clinical nurses. In this regard, Sabatino et al. (2016) have stated that the professional dignity of nurses increases when a supportive work environment is created and maintained in the policies of nursing managers [2]. Meanwhile, the clear and direct attention of nursing managers to the dignity of nurses is useful for achieving the goals of nurses and organizations. Accordingly, in healthy work environments where relationships between different professions are appropriate, nurses are likely not to leave their profession and are more interested in doing their work [51].

### Strengths & limitation

The present scale, however, was developed using a hybrid concept analysis approach. Also, unlike other studies, the present study conducts a thorough assessment of the psychometric properties (according to the COSMIN checklist), which is one of the strengths of the study. As well as, the results of the extraction factors in construct validity were consistent with the qualitative phase of study.

Some departments, especially those caring for COVID-19 patients, have had trouble cooperating due to the current COVID-19 outbreak and the increasing workload of clinical nurses. As a result, it is possible that data collection took longer than anticipated. Additionally, it is important to consider the potential limitations regarding the generalizability of the findings, as the samples were specifically selected from Iranian populations. Given that culture has been identified as a significant factor impacting nurses’ professional dignity, it may be beneficial to explore the applicability of this scale in other cultural contexts.

## Conclusions

The study findings indicated that PCNPDS consists of 22 items and is organized into three main dimensions: Organizational dignity, dignity-based competency, and dignity-based appreciation. The scale is deemed accurate, suitable, valid, reliable, and appropriate for the target population and can be utilized to assess professional dignity. Alternatively, exploring the efficacy of interventions aimed at enhancing this aspect among clinical nurses across various research studies may be worthwhile. Also, one of the most important applications of PCNPDS in nursing policies is to improve the status of nurses’ professional dignity. On the other hand, identifying and eliminating the factors that threaten the professional dignity of nurses with PCNPDS leads to the improvement of the workplace and the quality of life of nurses. This scale has several positive features, including a few items and user-friendly design, clear and easily understandable items, straightforward scoring, and a specific focus on measuring the professional dignity of clinical nurses.

### Electronic supplementary material

Below is the link to the electronic supplementary material.

Supplementary Material 1

Supplementary Material 2

## Data Availability

The datasets used and/or analyzed during the current study are available from the corresponding author on reasonable request.

## Abbreviations

PCNPDS: Perceived Clinical Nurses’ Professional Dignity Scale
WDS: Workplace Dignity Scale
CVR: Content validity ratio
CVI: Content validity index
I-CVI: Item content validity index
S-CVI: Scale content validity index
EFA: Exploratory factor analysis
CFA: Confirmatory factor analysis
ML: Maximum Likelihood
KMO: Kaiser-Meyer-Olkin
RMSEA: Root Mean Square of Error of Approximation
CFI: Comparative Fit Index
GFI: Goodness of Fit Index
AGFI: Adjusted Goodness of Fit Index
NFI: Normed Fit Index
PNFI: Parsimonious Normed Fit Index
IFI: Incremental Fit Index
ICC: Intraclass correlation coefficient
SEM: Standard Error of Measurement
MDC: Minimal Detectable Change
COSMIN: COnsensus-based Standards for the selection of health Measurement Instruments
