# Supplementary material for: Development and psychometric evaluation of perceived clinical nurses’ professional dignity scale: a sequential-exploratory mixed-method study
Source: BMC Nurs. 2023 Oct 20;22:397. doi: 10.1186/s12912-023-01543-y (PMC10588146; doi:10.1186/s12912-023-01543-y)
Supplement: Supplementary file 1 — Supplementary Material 1 [file 12912_2023_1543_MOESM1_ESM.pdf]

An example of the process of refining items based on overlapping items

	Overlapping items	Final items
1	Health care workers consider scientific position for me.	Health professionals consider scientific position for me.
2	My physician colleagues consider scientific position for me.	
3	My non-physician colleagues consider scientific position for me.	
4	I am treated with respect by patients.	I am treated with respect by patients and their families.
5	I am treated with respect by patients' families.	
6	I feel a sense of dignity due to the head nurse's appreciation at work.	I feel a sense of dignity due to the managers' appreciation.
7	I feel a sense of dignity due to the supervisor's appreciation at work.	
8	I feel a sense of dignity due to the matron's appreciation for taking proper care.	
9	Managers facilitate the essential conditions for continuing my education.	Managers facilitate the essential conditions for my professional development.
10	Managers facilitate the essential conditions for my continuous learning.	
11	Managers provide the necessary conditions for my development.	
12	As a nurse, I have a good position in	A nurse’s position is so important in

	**the hospital environment.**	**workplace.**
**13**	**The scientific position of nurses in the work environment is very important.**	
**14**	**As a nurse, I have an important role in workplace.**	
