# Supplementary material for: Development and psychometric evaluation of perceived clinical nurses’ professional dignity scale: a sequential-exploratory mixed-method study
Source: BMC Nurs. 2023 Oct 20;22:397. doi: 10.1186/s12912-023-01543-y (PMC10588146; doi:10.1186/s12912-023-01543-y)
Supplement: Supplementary file 2 — Supplementary Material 2 [file 12912_2023_1543_MOESM2_ESM.pdf]

Interview guide

The focus of the interview questions was the nurses' experience in the field of professional dignity, which was first a general question based on "When have you felt dignity or indignity in your profession?" It was asked and then the interviews were conducted following the questions according to the categories resulting from the literature review of and probing questions. We used such open questions as “What do you mean by this?” “Please elaborate on it”, and “Please give an example” to obtain more proper answers. In fact, the participants began to speak freely and by asking probing questions at the right time, we advanced the interview towards clarifying the phenomenon under study.

In this regard, some of the questions that were asked to the nurses are given below:

- What is the public image of nursing and what sense of professional dignity does it create in you?

- When you are respected and appreciated by others, what kind of professional dignity do you feel?

- When you are supported by managers, what sense of professional dignity do you feel?

- Do you think that proper communication with others makes you feel that your professional dignity has improved?

- If your dignity is improved, what are the consequences for you as a nurse?

- When your dignity is improved, what are the consequences for the patient and society?

- When your dignity is improved, what are the consequences for your organization?
